# Machine-learning for age-related macular degeneration using multimodal fundus data

**DOI:** 10.3389/fmed.2026.1756787

**Published:** 2026-03-31

**Authors:** Mengke Li, Aiping Gu, Hongyang Li, Yanying Li, Renlong Liang, Ting Su, Yi Wu

**Affiliations:** Department of Ophthalmology, The Affiliated Guangdong Second Provincial General Hospital of Jinan University, Guangzhou, Guangdong, China

**Keywords:** age-related macular degeneration, disease progression, machine learning, multimodal imaging, prediction model, random forest

## Abstract

**Objective:**

To develop an integrated model that combines multimodal fundus image features for accurately predicting the individualized risk of progression from early to late stages of age-related macular degeneration (AMD).

**Methods:**

A retrospective analysis was conducted on the data of 324 patients with AMD. The patients were randomly divided into a training set (*n* = 227) and a validation set (*n* = 97) at a ratio of 7:3. The follow-up period was 3 years, and patients with disease progression were defined as the progression group. In the training set, indicators related to prognosis were screened through univariate analysis. After variable compression by LASSO regression, independent influencing factors for poor prognosis were determined using multivariate logistic regression. Random Forest, Support Vector Machine, XG BOOST, and K-Nearest Neighbor algorithm prediction models were constructed using Python software. The performance of the models was evaluated by the area under the receiver operating characteristic curve (AUC), and the optimal model was selected.

**Results:**

There were no significant differences in the baseline characteristics between the training set and the validation set patients (*P* > 0.05), indicating comparability. In the training set, multivariate logistic regression analysis showed that pigmentary abnormalities, the total area of drusen in the macular area, and the ellipsoid zone were independent risk factors for disease progression (*P* < 0.05), while subfoveal choroidal thickness and choroidal capillary blood flow density were independent protective factors (*P* < 0.05). The AUC values of the Random Forest model (0.779 in the training set and 0.700 in the validation set) were significantly higher than those of the K-Nearest Neighbor algorithm (0.717 in the training set and 0.596 in the validation set), the Support Vector Machine model (0.768 in the training set and 0.646 in the validation set), and XG BOOST (0.702 in the training set and 0.762 in the validation set), making it the optimal prediction model.

**Conclusion:**

In this study, an AMD progression prediction model based on multimodal fundus images was successfully developed, which can effectively identify patients at high risk of progression and provide a new paradigm for clinical individualized precision medicine.

## Introduction

Age-related Macular Degeneration (AMD) is the leading cause of irreversible vision loss and severe visual impairment among the elderly worldwide (1). Its prevalence continues to rise with the intensification of the aging population, posing an increasingly severe public health challenge (2). The pathological process of AMD is usually divided into early, intermediate, and late stages. Early AMD is characterized by the formation of drusen and mild pigmentary abnormalities in the retinal pigment epithelium (RPE), and patients’ vision is mostly not significantly impaired (3). However, once the disease progresses to the late stage, it is manifested by choroidal neovascularization or geographic atrophy. At this time, photoreceptor cells undergo irreversible death, leading to severe and permanent loss of central vision (4). Therefore, the core challenge and critical window period in AMD management lie in the early stage of the disease process, that is, how to accurately identify those individuals at high risk of progressing to late-stage lesions in the next few years from a large number of early AMD patients with seemingly stable conditions.

Currently, in routine clinical practice, ophthalmologists mainly rely on the qualitative evaluation of color fundus photographs and the semi-quantitative grading based on the simplified severity scale of the Age-Related Eye Disease Study (AREDS) to judge the risk of AMD patients (5). However, this method largely depends on the individual experience of physicians, is highly subjective, and lacks precise quantitative standards, making it difficult to achieve refined and dynamic prediction of individual progression risk (6). More importantly, traditional assessment methods fail to fully integrate and utilize the massive, high-dimensional pathophysiological information provided by modern multimodal ophthalmic imaging techniques (7). Spectral domain optical coherence tomography can display the microscopic layered structures of the retina and choroid *in vivo* with unprecedented resolution and accurately quantify the volume of drusen, retinal thickness, and choroidal thickness. Optical coherence tomography angiography can non-invasively and clearly show the blood perfusion of the retinal and choroidal capillaries without injecting contrast agents (8). These multimodal imaging indicators reveal the pathological essence of AMD from different dimensions, including lipid metabolism disorders, retinal pigment epithelial dysfunction, thickening of Bruch’s membrane, choroidal vascular changes, and chronic inflammation. Therefore, the effective integration of this multi-source information will surely open up new ways for in-depth understanding of the disease progression mechanism and the construction of accurate prediction models (9). In recent years, several studies have developed AMD progression prediction models primarily based on single imaging modalities, such as color fundus photography or structural OCT. While these approaches have laid important foundations, they often overlook the functional dimension of disease pathophysiology (10). Notably, optical coherence tomography angiography (OCTA) provides a unique functional metric—choroidal capillary flow density—which directly reflects choroidal perfusion status. Choroidal ischemia is increasingly recognized as a key driver in AMD pathogenesis. By integrating this functional OCTA indicator with structural OCT and fundus photographic features, our model captures a more holistic view of the disease (11). Furthermore, we employ the Random Forest algorithm, which is particularly adept at modeling complex, non-linear interactions among multimodal features—a challenge for conventional statistical methods. Therefore, our study advances the field by fusing structural and functional imaging data within a robust machine learning framework, aiming to build a more biologically comprehensive and clinically actionable prediction tool.

In terms of data analysis methodology, traditional statistical models (such as Logistic regression) often struggle when dealing with the complex non-linear relationships and interactions between variables inherent in multimodal data. Machine learning, as the core branch of artificial intelligence, has algorithms that are particularly good at mining potential patterns from high-dimensional and complex data sets and constructing prediction models. Among them, the random forest algorithm has been widely used in medical prediction model research because of its outstanding advantages such as insensitivity to variable collinearity, ability to effectively handle mixed types of continuous and categorical variables, low susceptibility to overfitting, and built-in feature importance evaluation function, which can significantly improve the robustness and accuracy of the model.

Based on the above background, this study aims to meet a clear clinical need: to develop a practical tool for individualized and accurate prediction of the progression risk of early AMD. It is hypothesized that by systematically extracting and integrating key quantitative features from multimodal images such as color fundus photography, Optical Coherence Tomography (OCT), and Optical Coherence Tomography Angiography (OCTA), and using the powerful ensemble learning algorithm of random forest for modeling, an AMD progression prediction model with excellent performance and strong generalization ability can be constructed. The successful implementation of this study will not only provide a powerful decision-making support tool for the clinical management of AMD, promoting the transformation of the diagnosis and treatment model from “passive treatment” to “active prediction and prevention”, but also provide a valuable example for the application of multimodal data fusion and artificial intelligence technology in ophthalmology and other chronic disease fields.

## Materials and methods

### Study design and participants

This study was a retrospective longitudinal study. A total of 324 consecutive patients diagnosed with early AMD in the ophthalmology department of our hospital from January 2020 to January 2022 were enrolled. Inclusion criteria were as follows: (1) age ≥ 50 years; (2) meeting the internationally recognized diagnostic criteria for early AMD: the presence of medium-sized (diameter 63–125 μm) drusen, with or without RPE abnormalities (12); (3) completion of a complete multimodal imaging examination at baseline and during the follow-up period; (4) follow-up time ≥ 24 months. Exclusion criteria were as follows: (1) comorbid with other fundus diseases (such as diabetic retinopathy, retinal vein occlusion); (2) severe opacification of the refractive media affecting image quality; (3) a history of intraocular surgery (except cataract surgery); (4) incomplete follow-up data. This study was approved by the Ethics Committee of Guangdong Second Provincial General Hospital, with the approval number 2025-KY-KZ-535-01. All enrolled patients have signed written informed consent forms.

### Data collection

All patients completed the following standardized imaging examinations at the baseline: (1) Color fundus photography: A fundus camera (such as Canon CR-2) was used to take 45° field-of-view images centered on the macula; (2) Spectral domain OCT: A Spectralis OCT was used for linear and cubic scanning of the macular area, with a scanning range of 6 × 6 mm and 512 × 128 A-scans; (3) OCTA: An RTVue XR Avanti system was used for 3 × 3 mm macular area scanning, and images of the superficial capillary plexus, deep capillary plexus, and choroidal capillary layer were automatically segmented.

Pigmentary abnormalities were defined as the presence or absence of retinal pigment epithelial (RPE) pigmentary changes (including hyperpigmentation, hypopigmentation, or clumping) in the macular area, confirmed by combined evaluation of color fundus photographs and spectral domain OCT images. This variable was categorized as “Yes” (presence) or “No” (absence).

### Quantitative parameter measurement

All quantitative parameters were measured by two experienced ophthalmologists (blinded to patient outcomes) using dedicated matching imaging analysis software, including Spectralis OCT built-in software (Version 6.0, Heidelberg Engineering, Germany, 2019) and RTVue XR Avanti built-in software (Version 2020.1, Optovue, Inc., United States, 2020), with discrepancies resolved by consensus.

#### Drusen-related parameters

Total drusen area in the macular area (mm^2^): Measured using Spectralis OCT’s built-in segmentation software. The macular 6 × 6mm scan area was analyzed, and the software automatically segmented drusen (hyporeflective lesions located between the RPE and Bruch’s membrane) to calculate the total area.

Drusen volume in the macular area (mm^3^): Derived from the total drusen area and average drusen thickness (automatically calculated by the Spectralis OCT software) using the formula: Volume = Total drusen area × Average drusen thickness.

Proportion of soft drusen (%): Soft drusen were defined as drusen with reflectivity lower than the surrounding RPE (identified via combined OCT and color fundus photography). The number of soft drusen was counted, and the proportion was calculated as (Number of soft drusen / Total number of drusen) × 100%.

#### Retinal/choroidal structural parameters

Subfoveal choroidal thickness (μm): Measured manually using Spectralis OCT. The measurement point was the foveal center, and the distance from the outer border of the RPE to the inner border of the sclera was recorded.

Macular foveal retinal thickness (μm): Automatically calculated by Spectralis OCT’s segmentation software, representing the thickness of retinal tissue from the inner limiting membrane to the RPE at the foveal center.

Ellipsoid zone: Evaluated as a categorical variable (“ontinuous” or “Discontinuous”) based on OCT images. “Continuous” indicates the ellipsoid zone (located between the inner segment and outer segment of photoreceptors) presented as an uninterrupted hyperreflective band; “Discontinuous” indicates the band was interrupted or absent.

#### Blood flow density parameters

Superficial capillary plexus blood flow density (%): Measured using RTVue XR Avanti OCTA’s built-in analysis tool. The 3 × 3 mm macular scan area was segmented into the superficial capillary plexus (ranging from the inner limiting membrane to the outer plexiform layer). The software automatically calculated the proportion of perfused vessel area relative to the total scan area.

Deep capillary plexus blood flow density (%): Using the same RTVue XR Avanti OCTA system, the 3 × 3 mm macular scan area was segmented into the deep capillary plexus (ranging from the outer plexiform layer to the outer nuclear layer). The software automatically computed the ratio of perfused vessel area to the total scan area.

Choroidal capillary blood flow density (%): Utilizing the choroidal capillary layer segmentation module of the RTVue XR Avanti OCTA, the perfused vessel area in the 3 × 3 mm macular choroidal capillary layer was automatically calculated. The density was defined as the ratio of perfused area to the total scan area.

#### Other parameters

Macular foveal avascular zone (FAZ) area (mm^2^): Automatically measured by the FAZ analysis tool of the RTVue XR Avanti OCTA, representing the avascular area at the center of the superficial capillary plexus.

### Outcome definition

The interviewed patients were followed up every 6 months. The primary outcome was progression to intermediate or late-stage AMD. The specific grouping criteria were as follows: Progression group: Meeting any of the following criteria: (1) the new appearance of soft drusen of any size or a ≥ 25% increase in the proportion of soft drusen during follow-up; (2) the appearance of new or enlarged geographic atrophy; (3) the appearance of choroidal neovascularization confirmed by OCTA (13, 14); Non-progression group: Not meeting any of the above progression criteria during the entire follow-up period.

### Statistical analysis

To ensure a minimum of 5 events per predictor variable (EPV ≥ 5) and based on an estimated progression rate of 34% derived from the literature, we calculated that a minimum total sample size of approximately 74 subjects (25/0.34) was required. To account for approximately 20% data loss or exclusion, we increased the target sample size to 89 subjects (74 × 1.2). This study ultimately included 324 patients, far exceeding this minimum requirement and thereby ensuring the stability of the model-.

Statistical analysis was performed using SPSS 26.0 and R 4.2.3 software. Measurement data conforming to the normal distribution were expressed as x̄ ± s, and the *t*-test was used for comparison between groups. Measurement data not conforming to the normal distribution were expressed as median (inter-quartile range), and the Man-Whitney U test was used. Count data were expressed as the number of cases (percentage), and the χ^2^ test was used for comparison between groups. In the training set, univariate analysis was first performed to screen out indicators with *P* < 0.05. All parameters included in Tables 1, 2 are baseline data collected at patient enrollment. After variable compression by LASSO regression, multivariate logistic regression analysis was used to determine independent influencing factors. Random Forest, Support Vector Machine, XG Boost, and K-Nearest Neighbor algorithm models were constructed based on Python 3.8.5 software and the sklearn library. The Receiver Operating Characteristic (ROC) curve was drawn using GraphPad Prism 9.0.

**TABLE 1:** Comparison of baseline parameters (collected at enrollment) of patients in the training set and validation set.

Indicators		Training set (*n* = 227)	Validation set (*n* = 97)	*t/χ ^2^*	*P*
Age (years)		68.35 ± 8.47	67.94 ± 8.88	0.393	0.694
Gender	Male	124(54.63)	55(56.70)	0.118	0.731
	Female	103(45.37)	42(43.30)		
BMI (kg/m^2^)		25.72 ± 3.45	25.35 ± 3.61	0.872	0.384
Smoking history [n (%)]	Yes	98(43.17)	38(39.18)	0.446	0.504
	No	129(56.83)	59(60.82)		
Hypertension [n (%)]	Yes	112(49.34)	51(52.58)	0.285	0.593
	No	115(50.66)	46(47.42)		
Diabetes [n (%)]	Yes	55(24.23)	20(20.62)	0.498	0.480
	No	172(75.77)	77(79.38)		
Total cholesterol (mmol/L)		5.10 ± 0.91	5.04 ± 0.96	0.535	0.593
C-reactive protein (mg/L)		2.88 ± 1.78	2.73 ± 1.85	0.687	0.493
Drusen volume in the macular area (mm^3^)		0.04 ± 0.02	0.03 ± 0.02	1.455	0.147
Proportion of soft drusen (%)		25.45 ± 18.32	26.91 ± 19.15	0.648	0.517
Pigmentary abnormalities [n (%)]	Yes	95(41.85)	43(44.33)	0.171	0.679
	No	132(58.15)	54(55.67)		
Subfoveal choroidal thickness (μm)		199.12 ± 56.84	202.89 ± 60.35	0.537	0.592
Macular foveal retinal thickness (μm)		261.85 ± 34.67	259.14 ± 36.51	0.634	0.526
Total drusen area in the macular area (mm^2^)		0.18 ± 0.12	0.17 ± 0.11	0.704	0.482
Ellipsoid zone [n (%)]	Continuous	179(78.85)	74(76.29)	0.262	0.609
	Discontinuous	48(21.15)	23(23.71)		
Pigment epithelial detachment [n (%)]	Yes	41(18.06)	15(15.46)	0.321	0.571
	No	186(81.94)	82(84.54)		
Superficial capillary plexus blood flow density (%)		38.61 ± 4.79	39.08 ± 4.72	0.812	0.417
Deep capillary plexus blood flow density (%)		29.25 ± 5.19	28.80 ± 5.52	0.701	0.484
Choroidal capillary blood flow density (%)		51.41 ± 6.08	52.15 ± 5.95	1.010	0.313
Macular foveal avascular zone area (mm^2^)		0.29 ± 0.08	0.30 ± 0.07	1.069	0.286

**TABLE 2:** Univariate analysis of baseline influencing factors for progression events in early-stage AMD patients (all parameters were collected at enrollment).

Indicators		Progression group (*n* = 149)	Non-progression group (*n* = 78)	*t/*χ ^2^	*P*
Age (years)		67.51 ± 8.59	69.12 ± 8.68	1.336	0.183
Gender	Male	82(55.03)	45(57.69)	0.147	0.702
	Female	67(44.97)	33(42.31)		
BMI (kg/m^2^)		25.69 ± 3.41	25.91 ± 3.44	0.460	0.646
Smoking history [n (%)]	Yes	66(44.30)	36(46.15)	0.072	0.789
	No	83(55.70)	42(53.85)		
Hypertension [n (%)]	Yes	73(48.99)	45(57.69)	1.552	0.213
	No	76(51.01)	33(42.31)		
Diabetes [n (%)]	Yes	35(23.49)	23(29.49)	0.968	0.325
	No	114(76.51)	55(70.51)		
Total cholesterol (mmol/L)		5.11 ± 0.89	5.07 ± 0.92	0.318	0.751
C-reactive protein (mg/L)		2.89 ± 1.73	3.08 ± 1.80	0.775	0.439
Drusen volume in the macular area (mm^3^)		0.05 ± 0.04	0.04 ± 0.03	1.940	0.054
Proportion of soft drusen (%)		24.35 ± 18.11	27.92 ± 18.75	1.394	0.165
Pigmentary abnormalities [n (%)]	Yes	47(34.90)	46(57.69)	21.189	0.001
	No	102(65.10)	32(42.31)		
Subfoveal choroidal thickness (μm)		212.45 ± 53.27	192.18 ± 56.41	2.668	0.008
Macular foveal retinal thickness (μm)		263.84 ± 34.25	260.37 ± 35.29	0.717	0.474
Total drusen area in the macular area (mm^2^)		0.16 ± 0.10	0.22 ± 0.16	3.467	0.001
Ellipsoid zone [n (%)]	Continuous	131(84.56)	49(64.10)	14.343	0.001
	Discontinuous	18(15.44)	29(35.90)		
Pigment epithelial detachment [n (%)]	Yes	26(17.45)	20(25.64)	2.126	0.145
	No	123(82.55)	58(74.36)		
Superficial capillary plexus blood flow density (%)		39.15 ± 4.70	38.42 ± 4.88	1.097	0.274
Deep capillary plexus blood flow density (%)		29.78 ± 5.15	29.05 ± 5.36	1.000	0.318
Choroidal capillary blood flow density (%)		52.88 ± 5.52	50.15 ± 6.08	3.416	0.001
Macular foveal avascular zone area (mm^2^)		0.28 ± 0.07	0.29 ± 0.08	0.973	0.332

The “proportion of soft drusen (%)” reflects the baseline status of patients; progression criterion (1) refers to “new appearance or ≥ 25% increase in soft drusen during follow-up” (not baseline presence).

## Results

### Comparison of baseline data of patients in the training set and validation set

A total of 324 cases of early AMD were included in this study. According to the ratio of 7:3, 324 patients were divided into a training data set (including 227 cases) and a validation data set (including 97 cases). There were no statistically significant differences in the comparison of general data between patients in the training set and the validation set (*P* > 0.05) (Table 1).

### Univariate analysis of influencing factors for progression events in early-stage AMD patients

Univariate analysis showed that in the training set, there were statistically significant differences (*P* < 0.05) between the progression group and the non-progression group of patients in terms of pigmentary abnormalities, subfoveal choroidal thickness, total area of drusen in the macular area, ellipsoid zone, and choroidal capillary blood flow density (Table 2). Given the unequal variances observed in some continuous variables (e.g., total drusen area), a Mann-Whitney U test was also performed, confirming the significant difference between groups (*P* < 0.01).

### Multivariate logistic regression analysis of influencing factors leading to the progression of early-stage AMD patients

**FIGURE 1:**
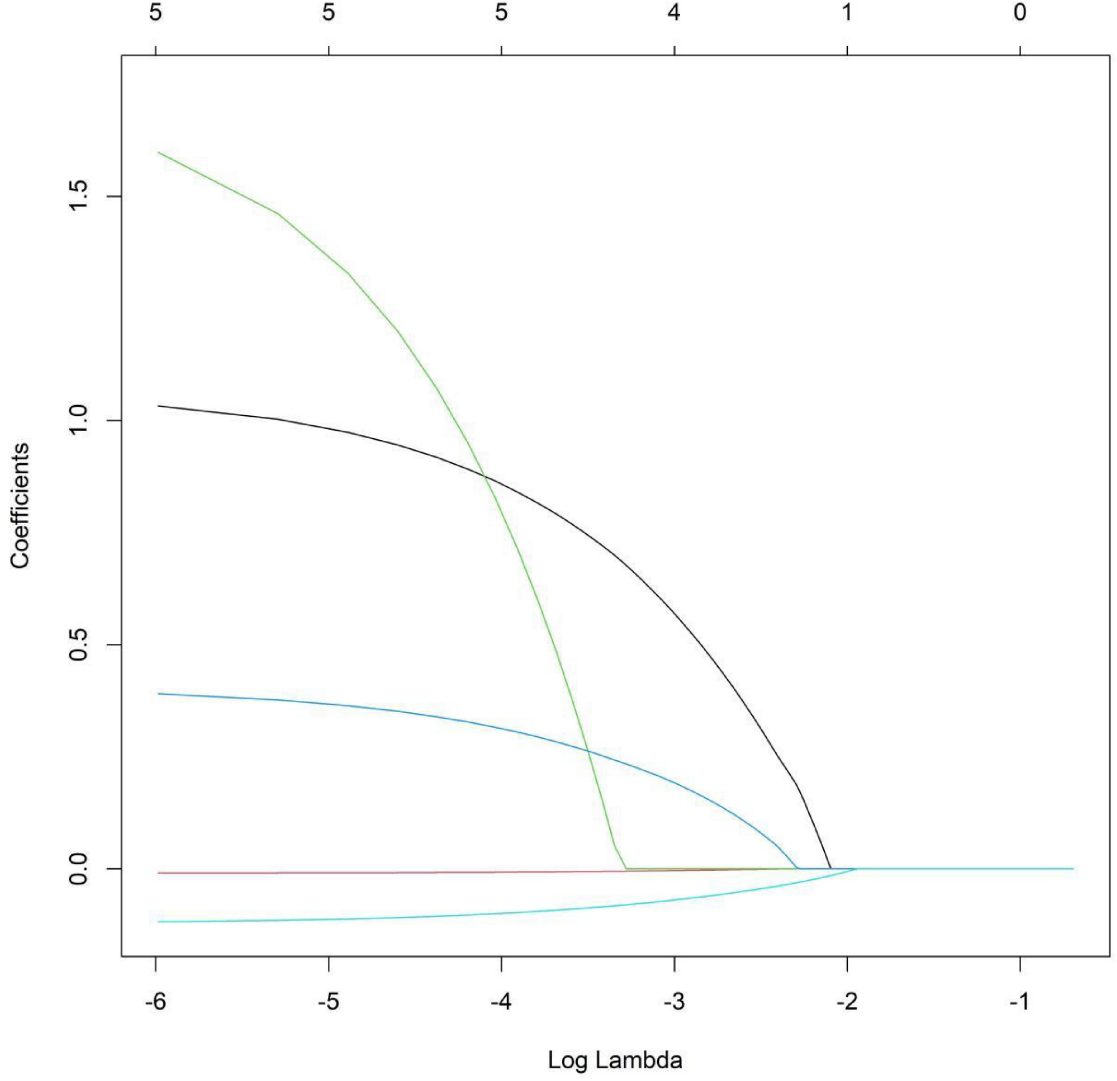
LASSO regression analysis diagram.

**FIGURE 2:**
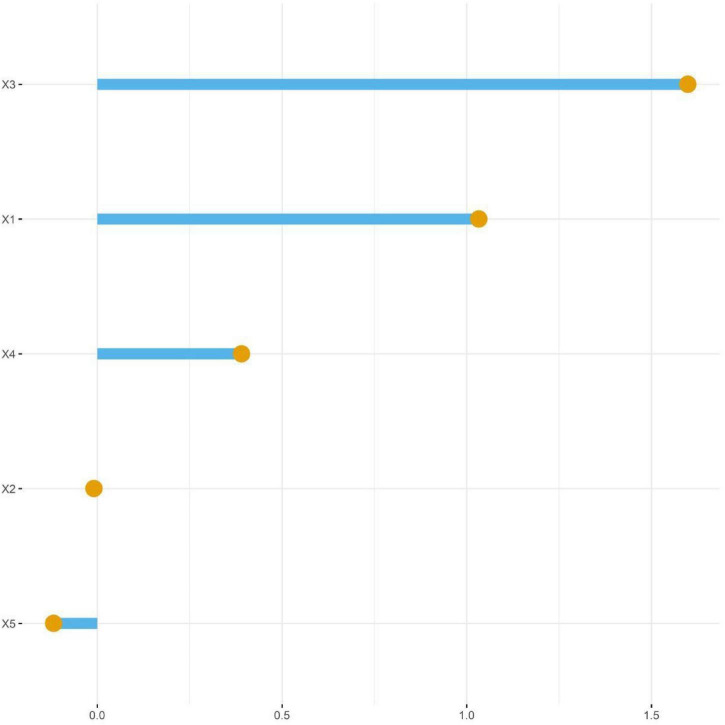
LASSO coefficient diagram (X1, Pigmentary abnormalities; X2, Subfoveal choroidal thickness; X3, Total area of drusen in the macular area; X4, Ellipsoid zone; X5, Choroidal capillary blood flow density).

Taking whether progression occurred as the dependent variable (progression group = 0, non-progression group = 1), the indicators with statistical significance in the univariate analysis were included in the LASSO regression for variable screening. Variables were selected using the screening criterion of lambda.1se (Figures 1, 2). The indicators with a relatively appropriate number of predictive variables were pigmentary abnormalities, subfoveal choroidal thickness, total area of drusen in the macular area, ellipsoid zone, and choroidal capillary blood flow density. The above indicators were included in the multivariate logistic regression analysis. The results showed that pigmentary abnormalities, total area of drusen in the macular area, and ellipsoid zone were independent risk factors for the progression of AMD patients, while subfoveal choroidal thickness and choroidal capillary blood flow density were independent protective factors for the progression of AMD patients (*P* < 0.05) (Table 3).

**TABLE 3:** Multivariate logistic regression analysis of influencing factors for the progression of early-stage AMD patients.

Indicators	β	SE	Wald	*P*	OR	95%CI
Pigmentary abnormalities	0.790	0.396	3.988	0.046	2.204	1.015–4.786
Subfoveal choroidal thickness	−0.009	0.003	9.752	0.002	0.991	0.985–0.997
Total area of drusen in the macular area	3.280	1.295	6.414	0.011	26.578	2.100–46.449
Ellipsoid zone	0.905	0.457	3.923	0.048	2.472	1.009–6.054
Choroidal capillary blood flow density	−0.089	0.028	10.105	0.001	0.915	0.866–0.966

To select the most predictive features for mechanical complications of degenerative scoliosis from high-dimensional candidate predictors and prevent model overfitting, LASSO (Least Absolute Shrinkage and Selection Operator) regression was used for feature selection in this study. Figure 1 shows the change paths of the coefficients of each predictor as the regularization parameter λ (after logarithmic transformation) increases.

As shown in Figures 1, 2, when the value of λ is small, the model complexity is high, and most features are retained. As the value of λ increases (i.e., the regularization intensity increases), the compression effect on the coefficients toward zero becomes more significant. At this threshold, the coefficients of 5 features are non-zero and are retained for subsequent model construction. These features include pigmentary abnormalities, subfoveal choroidal thickness, total area of drusen in the macular area, ellipsoid zone, and choroidal capillary blood flow density. The remaining features are automatically excluded by the model due to their weak predictive contribution.

### Prediction performance of machine-learning models in the training set and validation set

**FIGURE 3:**
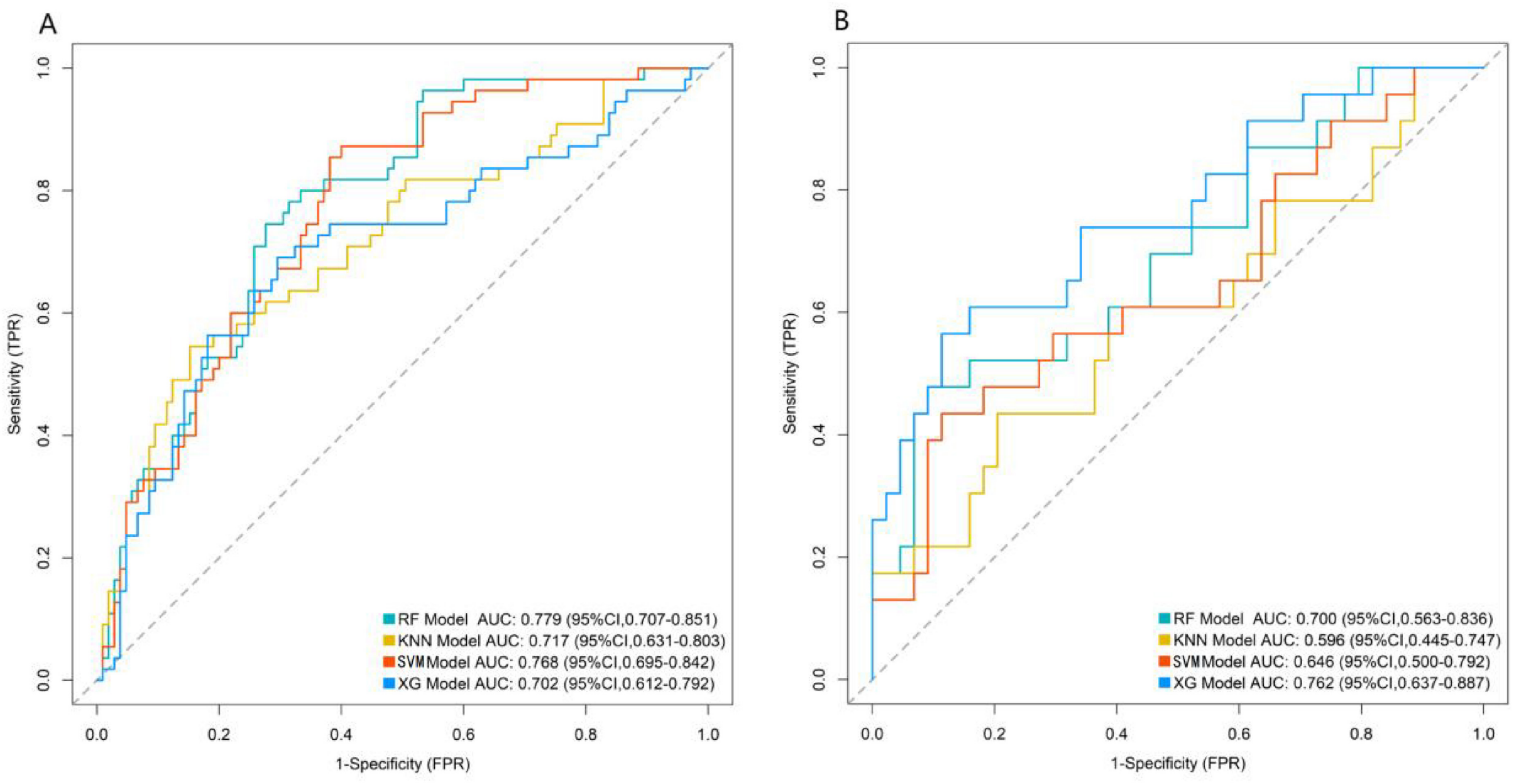
Area under the receiver operating characteristic curve of machine-learning models (**A:** Training set, **B:** Validation set).

The Random Forest model, K-Nearest Neighbors algorithm model, Support Vector Machine model, and XG BOOST model were used for prediction in the training set and validation set. The AUC values of the four models in the training set were 0.779, 0.717, 0.768, and 0.702, respectively, and the AUC values in the validation set were 0.700, 0.596, 0.646, and 0.762, respectively. The model with the largest AUC value was selected as the best model in this study, which was the Random Forest model (Figure 3).

### Interpretability evaluation of model prediction results

**FIGURE 4:**
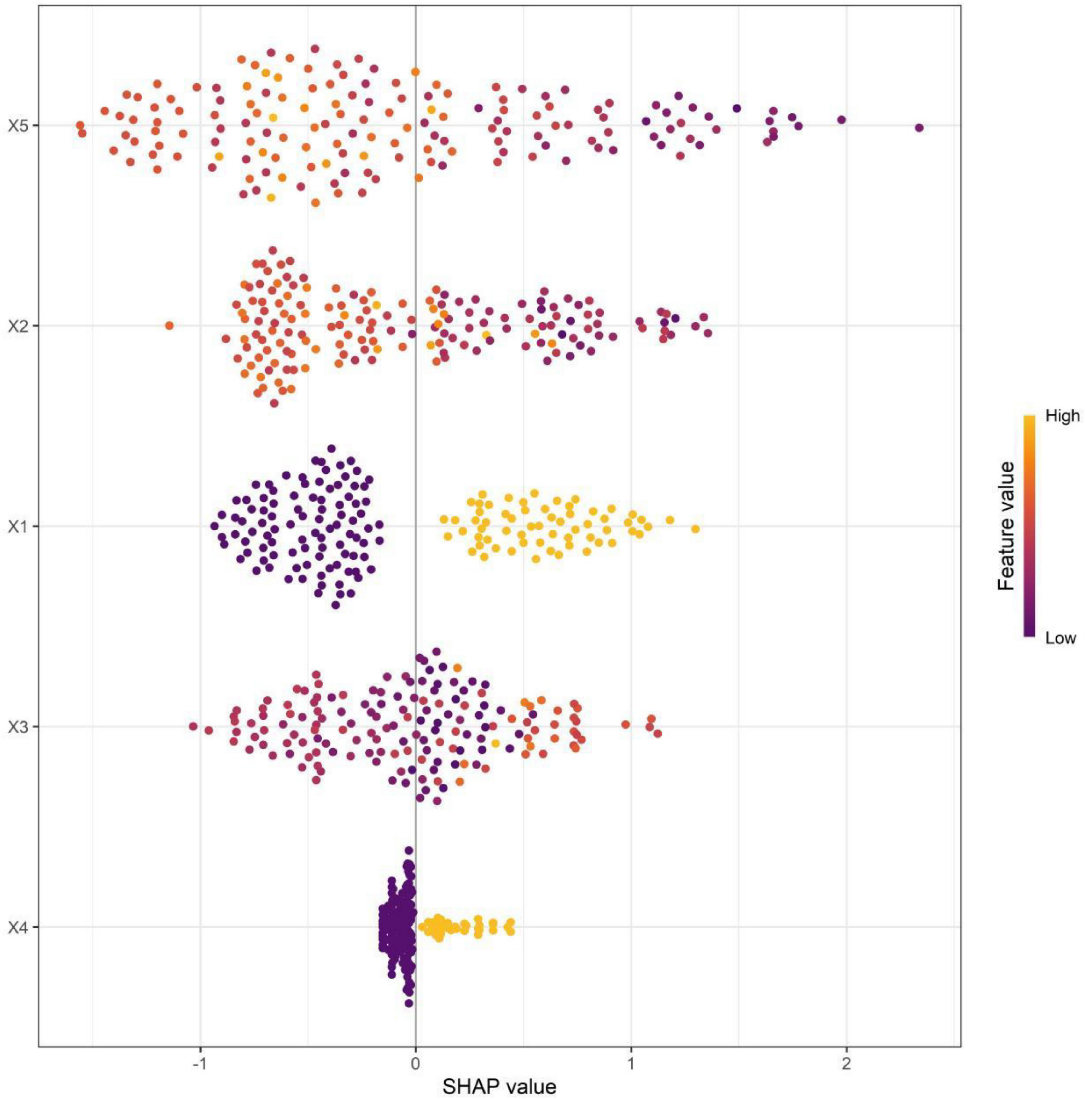
Variable importance in SHAP (X1, Pigmentary abnormalities; X2, Subfoveal choroidal thickness; X3, Total area of drusen in the macular area; X4, Ellipsoid zone; X5, Choroidal capillary blood flow density).

To enhance the clinical applicability and trustworthiness of the Random Forest model, we evaluated its interpretability using SHAP values. As shown in Figure 4, the SHAP plot intuitively reflects the direction and magnitude of each core predictor’s influence on AMD progression risk: for example, an increase in the total area of drusen in the macular area (a core risk factor) corresponds to a positive SHAP value, significantly elevating progression risk; in contrast, an increase in subfoveal choroidal thickness (a protective factor) corresponds to a negative SHAP value, reducing risk. This visualization allows clinicians to clearly understand which factors drive a specific patient’s risk score, thereby improving trust in the model’s predictions and facilitating its integration into clinical decision-making workflows. The horizontal axis represents the SHAP values, while the features on the vertical axis are sorted by their cumulative SHAP value influence. Each pointed data corresponds to a specific instance, and its position along the x-axis represents the SHAP value of that specific instance and feature. pigmentary abnormalities, subfoveal choroidal thickness, total area of drusen in the macular area, ellipsoid zone, and choroidal capillary blood flow density are the five most important factors for predicting whether AMD patients will experience disease progression. To intuitively compare the changes of core predictive indicators at baseline and the end of 3-year follow-up, the detection results of the five core indicators in the progression group and non-progression group at the final follow-up were compiled into Supplementary Table 1.

## Discussion

In this study, an integrated prediction model based on multimodal fundus images and the random forest algorithm was successfully developed and internally validated. The model demonstrated good discriminative ability in the training set (AUC = 0.779) and fair predictive performance in the validation set (AUC = 0.700), indicating potential clinical utility while acknowledging room for improvement. The results strongly confirm our initial hypothesis: by integrating quantitative indicators from different imaging modalities that reflect the core pathophysiological processes of AMD, a powerful prediction tool can be constructed, whose performance surpasses that of any single-modality or traditional risk score. The advantage of this model lies in its comprehensiveness. It not only captures the two classic markers for AMD diagnosis, drusen load and pigmentary abnormalities (15), but also integrates key information reflecting the choroidal structural support and microcirculation function, thus providing a more comprehensive biological biomarker profile regarding disease activity and progression risk.

The five core predictors retained in the random forest model each possess a strong biological rationale and clear clinical relevance. Among these, the total area of drusen in the macular region was identified as the most important predictor—a finding fully aligned with the long-standing understanding of drusen as the pathological hallmark of AMD. An increase in drusen area directly signals worsening metabolic disturbance and disease burden, serving as the most indicator of progression toward a more active disease stage (16). To bridge these predictors to clinical practice and enhance the interpretability—and thus the trustworthiness—of the model, we employed SHAP (Shapley Additive Explanations) analysis. As visualized in Figure 4, the SHAP summary plot illustrates the direction and magnitude of each predictor’s contribution to the estimated risk of AMD progression. For instance, a larger drusen area (a risk factor) corresponds to a positive SHAP value, elevating the predicted risk, whereas greater subfoveal choroidal thickness (a protective factor) corresponds to a negative SHAP value, lowering the risk. This individualized explanation allows clinicians to intuitively discern which factors drive a specific patient’s risk score, thereby facilitating trust in the model’s output and supporting its integration into clinical decision-making.

Second, the presence of pigmentary abnormalities intuitively reflects active cellular events such as dysfunction, death, migration, or proliferation of RPE cells under metabolic stress, and is an important clinical indicator of the disease being in the active stage (17). In addition, the discontinuity of the ellipsoid zone is an early and sensitive marker of mitochondrial dysfunction and structural damage in photoreceptor cells. Its appearance indicates that the pathological process of the disease has progressed from abnormalities in the RPE/Bruch’s membrane complex to affecting the functional units of the neural retina, and is a clear precursor of poor visual prognosis (18).

Finally, the choroidal capillary blood flow density and the subfoveal choroidal thickness respectively reveal the core role of the choroid in the progression of AMD from the functional and structural aspects (19, 20). The choroidal capillaries are the sole source of oxygen and nutrients for the outer retina (including RPE and photoreceptor cells). A decrease in its blood flow density directly leads to local ischemia, increased oxidative stress, and weakened ability to clear metabolic waste, thereby accelerating RPE cell apoptosis and photoreceptor damage. Notably, univariate analysis showed higher choroidal capillary blood flow density in the progression group, which initially seems contradictory to the ischemic theory. However, this can be explained by the stage-dependent dynamic changes of choroidal perfusion in AMD: In the early stage of disease progression, retinal metabolic stress may theoretically induce compensatory hyperperfusion of the choroidal capillary bed, which is a speculative view and needs to be verified by further longitudinal studies, leading to a potential temporary increase in blood flow density. This compensatory response precedes the progressive choroidal ischemia observed in advanced AMD, and thus does not contradict the long-term ischemic mechanism of AMD. Additionally, the measurement was limited to the macular 3 × 3 mm area, and regional perfusion variations may not fully represent global choroidal ischemia. Multivariate logistic regression further confirmed choroidal capillary blood flow density as a protective factor, indicating that after adjusting for other confounders, higher baseline blood flow density is associated with a lower progression risk—consistent with the core of the ischemic theory. The thinning of the choroidal thickness may reflect choroidal vascular atrophy, fibrosis, or chronic inflammatory infiltration, and is a macroscopic structural manifestation of the “weakening” of the supporting tissue. The inclusion of these two indicators highlights the decisive role of the choroidal health status in the occurrence and development of AMD, extending the model’s insight from the retinal level to its underlying suitable environment.

Comparing the performance of this model with the existing literature can highlight its value. Some previous studies attempted to use single-OCT or color fundus photography features for prediction, and their AUC values were generally between 0.70 and 0.80 (21). A few cutting-edge studies began to attempt to fuse multimodal data, but most were limited to the quantitative features of OCT. This study improved the discriminative ability of the model by introducing the unique functional indicator of choroidal capillary blood flow density provided by OCTA. This finding echoes the theory emphasized in recent basic research on the important role of vascular factors and ischemia in the pathology of AMD, demonstrating the great potential of functional imaging in prediction models (22). The AUC of this model reaches 0.779, which is in the upper range of the performance of currently reported models, fully demonstrating the effectiveness of the systematic multimodal data fusion strategy.

In choosing the random forest as the core algorithm, this study also shows full consideration. Compared with traditional Logistic regression, the random forest can automatically handle the complex non-linear relationship between predictors and outcomes without the need for researchers to perform complex variable transformations in advance. For example, the relationship between drusen area and progression risk may not be a simple linear one, and there may be a “threshold effect” (23), which the random forest can capture well. At the same time, the built-in Bootstrap resampling and random feature subspace selection mechanisms of the algorithm endow the model with strong anti-overfitting ability, ensuring its robust performance on unknown data (validation set). In addition, the Gini importance output provided by the random forest not only enhances the interpretability of the model, enabling us to identify the core indicators with the most clinical value, but also points the way for future further simplification of the model or development of bedside risk assessment tools.

The successful development of this prediction model has far-reaching prospects for clinical translation. First, it can achieve true risk-stratified management. For patients judged to be at high risk by the model, clinicians can adopt more aggressive intervention strategies, such as shortening the follow-up interval to 3–6 months, strengthening education on home Amsler grid monitoring (24), and even when approved ophthalmic drugs are available in the future, giving priority to including them in preventive treatment clinical trials. Conversely, for low-risk patients, the follow-up cycle can be appropriately extended to 1 year, reducing their medical burden and anxiety, and achieving optimal allocation of medical resources (25). Second, the model can serve as an effective tool for patient education and communication. Through visualizing the risk probability, it helps patients understand their own condition more intuitively, thereby improving treatment compliance and self-management ability. From a broader perspective, this study provides a specific example for the practice of precision medicine in the ophthalmic field, marking that the management of AMD is moving from a one-size-fits-all model based on population epidemiological data to a specialized and targeted new era based on individual multimodal biomarkers.

However, the limitations of this study must be objectively acknowledged. First, this is a single-center retrospective study, which inherently relies on existing medical records and may introduce selection bias and confounding factors. Although rigorous internal validation was performed, the model’s performance lacks external validation—its universality and robustness need to be further verified in multi-center cohorts involving different regions, diverse populations, and various brands of imaging equipment. Therefore, future work should prioritize external validation in multi-center, prospective cohorts with heterogeneous populations and imaging devices to enhance its generalizability. Second, the total sample size of 324 patients is relatively modest for machine learning modeling, and the validation set (*n* = 97) in particular may not fully capture the diversity of the general population, which could limit the model’s external validity. Future studies will seek to expand the sample size, especially by including more diverse patient cohorts in multi-center settings, to further validate and enhance the model’s generalizability. Third, the optimal Random Forest model achieved an AUC of 0.700 in the validation set, which is generally considered “fair” rather than “excellent” in clinical prediction models. This indicates that there is still a significant margin of error in the model’s predictive performance, and its accuracy needs further improvement. Future studies will optimize the model through strategies such as expanding the sample size, integrating multi-dimensional biomarkers, and refining algorithm parameters to enhance its discriminative ability. Fourth, as AMD is a chronic, lifelong disease, the 3-year follow-up window in this study may miss late-stage progressions that occur over a longer timeframe; a more extended follow-up (5–10 years) is needed to evaluate the model’s ability to predict long-term disease progression. Fifth, all predictors included in this study are imaging indicators. To construct a more comprehensive “panoramic” prediction model and further improve accuracy, future studies will expand the sample size through multi-center collaboration, extend the follow-up period to 5–10 years, and integrate additional biomarkers such as genetic information (e.g., CFH, ARMS2 gene polymorphisms) and serum indicators (e.g., complement factors, inflammatory factors). Sixth, this study did not conduct a stratified analysis of vascular changes based on the disease stage of AMD (advanced vs. intermediate), which makes it impossible to clarify the dynamic changes of choroidal blood perfusion in different stages of AMD progression, and also limits the in-depth analysis of the relationship between choroidal vascular characteristics and disease progression stage. Seventh, although the Random Forest model demonstrated the best performance, it is inherently a “black-box” algorithm. While feature importance analysis has provided some interpretability, its decision-making path is less intuitive compared to linear models. Future research will explore interpretability tools such as SHAP (SHapley Additive exPlanations) and LIME (Local Interpretable Model-agnostic Explanations) to provide clear attribution analysis for individual patient risk scores, facilitating clinical trust and application. Finally, during initial feature screening, we considered potential markers such as reticular pseudodrusen and intraretinal hyperreflective foci (IHRF) (26); however, these failed to reach statistical significance in univariate analysis and were not included in the final model. Larger-sample studies in the future may help validate the predictive value of these markers.

## Data Availability

The original contributions presented in the study are included in the article/Supplementary material, further inquiries can be directed to the corresponding authors.
