# Supplementary material for: Machine-learning for age-related macular degeneration using multimodal fundus data
Source: Front Med (Lausanne). 2026 Mar 31;13:1756787. doi: 10.3389/fmed.2026.1756787 (PMC13076296; doi:10.3389/fmed.2026.1756787)
Supplement: Supplementary file 1 [file Table_1.docx]

**Supplementary Table 1** Summary of 3-year follow-up results for patients with early age-related macular degeneration

| Indicators | Progression group (n=149) | | Non-progression group (n=78) | *t/χ²* | *P* |
| --- | --- | --- | --- | --- | --- |
| Pigmentary abnormalities [n (%)] | Present:112(75.17)  Absent:37(24.83) | | Present:30(38.46)  Absent:48(61.54) | 32.156 | 0.001 |
| Subfoveal choroidal thickness (μm, xˉ±s) | 176.23±48.56 | | 208.95±52.31 | 4.982 | 0.001 |
| Total drusen area in the macular area (mm², xˉ±s) | 0.29±0.15 | | 0.15±0.09 | 8.765 | 0.001 |
| Ellipsoid zone [n (%)] | Continuous:65(43.62)  Discontinuous:84(56.38) | Continuous:68(87.18)  Discontinuous:10(12.82) | | 40.219 | 0.001 |
| Choroidal capillary blood flow density (%, xˉ±s) | 46.32±5.89 | 53.68±6.12 | | 8.257 | 0.001 |

**Note：**This table presents the 3-year follow-up results of core independent influencing factors for early AMD progression in the training set, with measurement data expressed as mean ± standard deviation (xˉ±s) and analyzed by t-test, count data as n (%) and analyzed by *χ²* test, all indicators were detected with the same equipment and methods as baseline, and the results were determined by two experienced ophthalmologists blinded to patient outcomes to ensure the consistency and comparability with baseline data.
